# Cardiotoxicity as indicated by LVEF and troponin T sensitivity following two anthracycline-based regimens in lymphoma: Results from a randomized prospective clinical trial

**DOI:** 10.18632/oncotarget.8685

**Published:** 2016-04-11

**Authors:** Kai Xue, Juan J. Gu, Qunling Zhang, Xiaojian Liu, Jiachen Wang, Xiao-qiu Li, Jianfeng Luo, Francisco J. Hernandez, Stanley F. Fernandez, Myron S. Czuczman, Junning Cao, Xiaonan Hong, Ye Guo

**Affiliations:** ^1^ Department of Medical Oncology, Fudan University Shanghai Cancer Center, Shanghai, China; ^2^ Department of Oncology, Shanghai Medical College, Fudan University, Shanghai, China; ^3^ Department of Medicine & Immunology, Roswell Park Cancer Institute, Buffalo, NY, USA; ^4^ Department of Pathology, Fudan University Shanghai Cancer Center, Shanghai, China; ^5^ Department of Biostatistics, School of Public Health, Fudan University, Shanghai, China; ^6^ Department of Medicine, University at Buffalo, the State University of New York, Buffalo, NY, USA

**Keywords:** cardiac toxicity, doxorubicin, epirubicin, high-sensitivity troponin T

## Abstract

Anthracycline-induced cardiotoxicity influences treatment selection and may negatively affect clinical outcomes in lymphoma patients. While epirubicin induced cardiotoxicity less often than the same dose of doxorubicin in breast cancer, higher doses of epirubicin are required in lymphoma regimens for equivalent efficacy. Whether a higher dosage of epirubicin also induces cardiotoxicity less often than doxorubicin in lymphoma remains unknown. We therefore administered 6-8 cycles of cyclophosphamide, vincristine and prednisone (CEpOP) +/− rituximab (R) with either epirubicin (CEpOP) or doxorubicin (CHOP) to patients (N=398) with untreated diffuse large B-cell lymphoma (DLBCL) or follicular lymphoma grade 3 (FLG3). Left ventricular ejection fraction (LVEF) and high-sensitivity serum cardiac troponin T (HsTnT) were assessed at baseline and after 4 cycles of treatment. Epirubicin (70 mg/m^2^/dose) was equivalent to doxorubicin (50 mg/m^2^/dose) in terms of 3-year progression-free survival. The risk of decreased LVEF was similar between the two regimens. CEpOP+/−R induced HsTnT elevation less often than CHOP+/−R. We conclude that CEpOP+/−R is a more acceptable regimen with short-term efficacy similar to CHOP+/−R in lymphoma patients. Longer follow-up is needed to monitor the risk of cardiac dysfunction and determine whether differences in the induction of elevated HsTnT between epirubicin and doxorubicin justify changes in clinical practice.

## INTRODUCTION

Diffuse large B-cell lymphoma (DLBCL) and follicular lymphoma (FL) are two commonly diagnosed subtypes of Non-Hodgkin's Lymphoma (NHL), representing about 40% and 5% of new NHL cases annually in the People's Republic of China, respectively [1, 2]. Based on the number of centroblasts (cb) per high-power field (cb/HPF), FL can be pathologically subcategorized as grade-1 (0-5cb/HPF), grade-2 (6-15cb/HPF), or grade-3a (>15cb/HPF with centrocytes)/3b (>15cb/HPF in sheets with minimal numbers of centrocytes). The clinical behavior of FL grade 3b (FLG3b) is similar to DLBCL, and both are often managed with the same therapeutic approach [3].

Rituximab in combination with chemotherapy has become integral in the management of previously untreated DLBCL and in specific clinical subsets of FL (i.e. FLG3b). Anthracycline-based polychemotherapy (i.e. cyclophosphamide, doxorubicin, vincristine, and prednisone [CHOP]), combined with rituximab (R-CHOP), is the preferred front-line treatment in DLBCL and FLG3b.

Anthracycline-related cardiac toxicity is a serious complication that hinders the clinical outcomes and/or quality of life of cancer patients. However, DLBCL patients treated with non-anthracycline front-line chemotherapy regimens exhibit inferior overall response rates (ORR), progression free survival (PFS), and overall survival (OS), and anthracycline drugs thus play a pivotal role in the management of aggressive B-cell lymphomas. The incidence of doxorubicin-related cardiac toxicity ranges between 5-26% and influences treatment selection for certain patient populations in the clinical setting, including elderly patients and those with multiple co-morbid conditions or previous history of coronary artery disease [4].

Epirubicin is a 4′hydroxyl epi-isomer of doxorubicin with a more favorable toxicity profile. The incidence of cardiac toxicity and/or reduction in the left ventricular ejection fraction (LVEF) among breast cancer patients treated with epirubicin is lower than in patients treated with doxorubicin at equivalent cumulative dose schedules [5]. To achieve equivalent efficacy in lymphoma patients, a higher dosage of epirubicin has been used compared to doxorubicin (70mg/m^2^/dose vs. 50mg/m^2^/dose, respectively) [6, 7]. However, it is still unclear whether a higher dosage of epirubicin induces cardiotoxicity less often than doxorubicin.

Clinically, Schwartz *et al.* observed a direct correlation between post-chemotherapy decrease in LVEF and increased risk of chronic heart failure (CHF) following doxorubicin-based chemotherapy; this served as the basis for the development of standard guidelines to monitor LVEF changes during and/or after chemotherapy [8]. The assessment of LVEF by a multi-gated acquisition (MUGA) scan is objective and easily reproducible, and is therefore the preferred method for evaluating anthracycline-induced cardiac toxicity at our institution [9]. Although current cardiac imaging studies can assess heart structure and function during and after chemotherapy, changes are only detected once significant heart damage has occurred. Thus, there is a need to identify, test, and validate novel biomarkers that predict early heart damage in cancer patients receiving cardiotoxic agents. Elevated serum troponin levels were reported to detect cardiac cell damage and irreversible cardiomyocyte necrosis [10-17]. Troponin is a complex of three regulatory proteins (troponin C, troponin I, and troponin T [TnT]) that are involved in skeletal and cardiac muscle contraction. During the administration of systemic chemotherapy, detectable increases in TnT were associated with abnormally reduced left ventricular (LV) mass and LV end-diastolic posterior wall thickness as evaluated by long-term echocardiogram [18].

In an attempt to evaluate an alternative anthracycline regimen for the management of FLG3 and DLBCL, we conducted a prospective phase III clinical trial comparing epirubicin- and doxorubicin-based chemotherapy regimens with or without rituximab in lymphoma patients. We found there was no difference between these two groups in terms of incidence of decreased LVEF; these data have been presented in part at the 55th Annual Meeting of the American Society of Hematology, New Orleans 2013 [19]. In the present work, we report the final results regarding differences in both LVEF and serum troponin between groups of lymphoma patients treated with either epirubicin or doxorubicin.

## RESULTS

**Figure 1:**
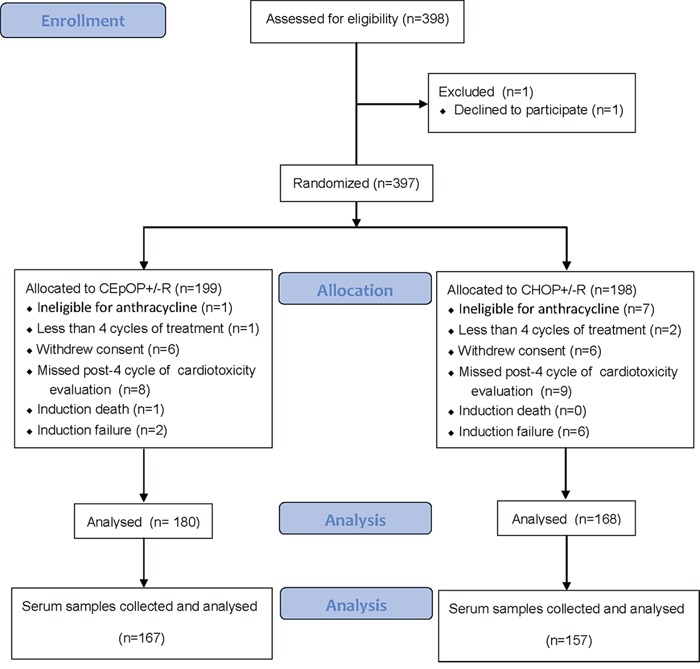
Patient distribution CONSORT diagram showing the allocation and disposition of patients with diffuse large B cell lymphoma (DLBCL) and follicular lymphoma grade 3 (FLG3) treated under this randomized controlled clinical trial (NCT00854568).

Between March 2009 and December 2012, a total of 398 patients were randomly assigned to either the CEpOP/R+CEpOP (N=199, 67/132) or CHOP/R+CHOP (N= 198, 56/142) groups. A total of 50 patients were excluded from the final analysis for the following reasons: refused random treatment assignment (N=1), were ineligible for anthracycline-based chemotherapy (N=8), completed < 4 cycles of the planed treatment due to non-cardiac toxicity (N=3), withdrawal of consent (N=12), missing LVEF after the 4^th^ cycle of treatment (N=17), early death during initial therapy (N=1), or primary refractory disease (N=8). Data from all other patients was analyzed (N=348, CEpOP+/−R=180, CHOP+/−R=168). Serum for HsTnT assays was collected from 324 patients, or 167 (92.8%) of those in the CEpOP+/−R group and 157 (93.5%) of those in the CHOP+/−R group. Figure 1 illustrates the clinical trial treatment information and patient distribution. Epidemiological, clinical, and pathological characteristics are described in Table 1. In general, the baseline demographic and clinical characteristics that could have influenced the occurrence of cardiac toxicity and/or changes in LEVF during therapy were equally distributed among treatment groups.

**Table 1:** Baseline demographic and clinical characteristics of 348 analyzed patients (324 patients with serum collected) with DLBCL or FLG3 treated with CEpOP+/−R or CHOP+/−R

	CEpOP+/−R	CHOP+/−R	CEpOP+/−R (serum collected)	CHOP+/−R (serum collected)
Characteristics	No. (%)	No. (%)	No. (%)	No. (%)
No. of patients	180	168	167	157
Age at diagnosis, median (range, years)	53 (19-75)	52 (19-74)	53 (19-75)	50 (19-74)
>65	15 (8.3)	13 (7.7)	14 (8.4)	12 (7.6)
18-65	165 (91.7)	155 (92.3)	153 (91.6)	145 (92.4)
Sex				
Male	93 (51.7)	90 (53.6)	87 (52.1)	85 (54.1)
Female	87 (48.3)	78 (46.4)	80 (47.9)	72 (45.9)
Concomitant diseases				
Yes	32 (17.8)	34 (20.2)	30 (18.0)	30 (19.1)
No	148 (82.2)	134 (79.8)	137 (82.0)	127 (80.9)
Hypertension				
Yes	27 (15.0)	27 (16.1)	26 (15.6)	24 (15.3)
No	153 (85.0)	141 (83.9)	141 (84.4)	133 (84.7)
ACEI and/or beta-blocker				
Yes	5 (2.8)	6 (3.6)	6 (3.6)	5 (3.2)
No	23 (85.2)	21 (77.8)	161 (96.4)	152 (96.8)
Diabetes mellitus				
Yes	7 (3.9)	12 (7.1)	6 (3.6)	8 (5.1)
No	173 (96.1)	156 (92.9)	161 (96.4)	149 (94.9)
Heart disease				
Yes	3 (1.7)	5 (3.0)	3 (1.8)	4 (2.5)
No	177 (98.3)	163 (97.0)	164 (98.2)	153 (97.5)
Smoking status				
Yes	43 (23.9)	37 (22.0)	40 (24.0)	35 (22.3)
No	137 (76.1)	131 (78.0)	127 (76.0)	122 (77.7)
BMI (mean ± Std, Kg/m^2^)	23.4 ± 2.8	23.3 ± 3.1	23.2 ± 2.8	23.4 ± 3.0
<18.5	8	6	7	6
18.5-23.9	98	100	95	94
24-26.9	57	46	53	41
>26.9	17	16	12	16
LVEF (mean ± Std, %)	67.0 ± 6.0	66.7 ± 6.9	67.0 ± 6.0	67.1 ± 6.8
Hemoglobin (mean ± Std, g/L)	132.5 ± 16.2	132.5 ± 17.2	132.5 ± 16.5	132.0 ± 17.2

Abbreviations: DLBCL = diffuse large B-cell lymphoma; FLG3 = follicular lymphoma grade 3; CEpOP+/−R = cyclophosphamide, epirubicin, vincristine and prednisone +/− rituximab; CHOP+/−R = cyclophosphamide, doxorubicin, vincristine and prednisone +/− rituximab; ACEI = angiotensin converting enzyme inhibitor; LVEF = left ventricular ejection fraction; BMI = body mass index.

### Anti-tumor activity and adverse events

**Figure 2:**
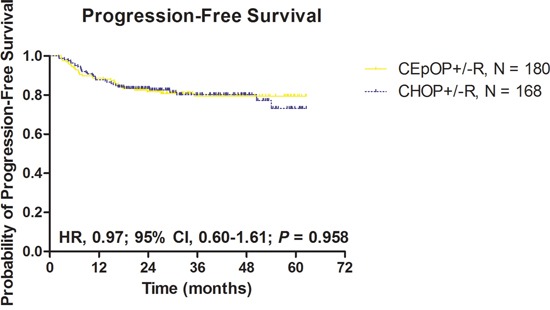
Progression-free survival according to treatment group: CEpOP +/−R vs. CHOP+/−R Abbreviations: CEpOP: Cyclophosphamide epirubicin, vincristine, and prednisone or cyclophosphamide; CHOP: cyclophosphamide, doxorubicin, vincristine, and prednisone; and R: rituximab

With a median follow-up of 27.1 months, ORR, CR rate, and PFS did differ between the CHOP+/−R and CEpOP+/−R groups after adjustment for histology subtype (DLBCL vs. FLG3) and rituximab use (Table 2, Figure 2). In addition, non-hematological toxicity was similar in patients treated with CHOP+/−R or CEpOP+/−R. The incidence of febrile neutropenia was higher among patients treated with CHOP+/−R compared to CEpOP+/−R (27.4% vs. 17.2% respectively, *P* = 0.023) (Table 2).

**Table 2:** Clinical activity and adverse events

	CEpOP+/−R*	CHOP+/−R*	
	No. (%)	No. (%)	*P* value
No. of patients	180	168	
Histology			0.733
DLBCL	162 (90.0)	153 (91.1)	
FLG3	18 (10.0)	15 (8.9)	
Rituximab combination	126 (70.0)	122 (72.6)	0.678
Median cycles (range)	6 (4-8)	6 (2-8)	0.588
Dose reduction	42 (23.3)	44 (26.2)	0.537
Reduction once	33 (18.3)	37 (22.2)	0.391
Reduction twice	9 (5.0)	7 (4.2)	0.711
Therapeutic effects			
DLBCL	162	153	
Molecular subtypes			0.760
GCB	57 (35.2)	48 (31.4)	
Non-GCB	87 (53.7)	88 (57.5)	
Unavailable/not assessable	18 (11.1)	17 (11.1)	
Stage			0.839
I	51 (31.5)	55 (35.9)	
II	65 (40.1)	59 (38.6)	
III	32 (19.8)	26 (17.0)	
IV	14 (8.6)	13 (8.0)	
IPI score			0.435
0	90 (55.6)	90 (58.8)	
1	44 (27.2)	41 (26.8)	
2	23 (14.2)	14 (9.2)	
3	5 (3.1)	8 (5.2)	
Chemotherapy+R	113	111	
CR/CRu	86 (76.1)	77 (69.4)	0.257
CR/CRu+PR	111 (98.2)	106 (95.5)	0.240
Estimated 3-year PFS (%)	83.3	82.4	0.943
Chemotherapy	49	42	
CR/CRu	30 (61.2)	26 (61.9)	0.947
CR/CRu+PR	46 (93.9)	41 (97.6)	0.385
Estimated 3-year PFS (%)	75.1	78.0	0.845
FLG3	18	15	
Subtypes			0.157
FLG3a	6	10	
FLG3b	4	2	
Unavailable/not assessable	8	3	
Stage			0.525
I	5 (27.8)	5 (33.3)	
II	5 (27.8)	6 (40.0)	
III	6 (33.3)	4 (26.7)	
IV	2 (11.1)	0 (0.0)	
IPI score			0.115
1	7 (38.9)	11 (73.3)	
2	6 (33.3)	3 (20.0)	
3	5 (27.8)	1 (6.7)	
Chemotherapy+R	13	11	
CR/CRu	8 (61.5)	10 (90.1)	0.098
CR/CRu+PR	12 (92.3)	11 (100.0)	0.347
Estimated 3-year PFS (%)	74.0	74.1	0.660
Chemotherapy	5	4	
CR/CRu	2 (40.0)	2(50.0)	0.764
CR/CRu+PR	4 (80.0)	3 (75.0)	0.858
Estimated 3-year PFS (%)	50.0	75.0	0.282
Adverse Events			
3/4 neutropenia	134 (74.4)	137 (81.5)	0.111
Febrile neutropenia	31 (17.2)	46 (27.4)	0.023
3/4 thrombocytopenia	2 (1.1)	0 (0.0)	0.171
3/4 Liver function	2 (1.1)	3 (1.8)	0.597
3/4 Renal function	0 (0.0)	0 (0.0)	NA

Abbreviation: CEpOP+/−R = cyclophosphamide, epirubicin, vincristine and prednisone +/− rituximab; CHOP+/−R = cyclophosphamide, doxorubicin, vincristine and prednisone +/− rituximab; R = rituximab; DLBCL = diffuse large B-cell lymphoma; FLG3 = follicular lymphoma grade 3; GCB = germinal center B-cell-like; IPI = international prognostic index; CR = complete remission; CRu = unconfirmed complete remission; PR = partial response; PFS = progression free survival.

* Percentages denote comparative proportions between the two different chemotherapy groups.

### Decreased LVEF in DLBCL/FLG3 patients treated with CHOP+/−R or CEpOP+/−R

**Figure 3:**
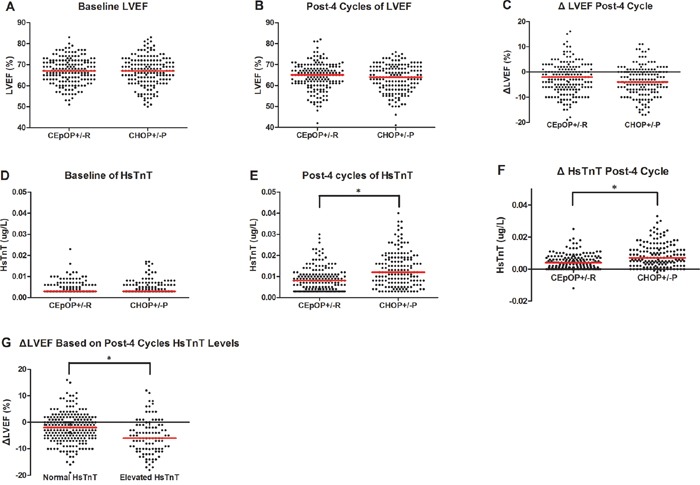
Imaging (LVEF by MUGA scan) and serum (HsTnT) cardiac monitoring during therapy in patients treated with CEpOP +/−R vs. CHOP+/−R Data from the CEpOP+/−R group is on the left, and data from the CHOP+/−R group is on the right. Red vertical bars represent the median. **A.** Baseline LVEF differences in patients treated with CEpOP+/−R and CHOP+/−R. **B.** LVEF differences in patients treated with CEpOP+/−R and CHOP+/−R after 4 cycles of therapy. **C.** Absolute decreases in LVEF in patients treated with CEpOP+/−R and CHOP+/−R after 4 cycles of chemotherapy. **D.** Baseline HsTnT differences in patients treated with CEpOP+/−R and CHOP+/−R. **E.** HsTnT differences in patients treated with CEpOP+/−R and CHOP+/−R after 4 cycles of treatment, *(*P* < 0.001). **F.** Absolute increases in HsTnT levels in patients treated with CEpOP+/−R and CHOP+/−R after 4 cycles of chemotherapy, *(*P* = 0.001). **G.** Absolute decreases in LVEF in patients with elevated and normal HsTnT levels after 4 cycles of treatment, *(*P* = 0.009). Abbreviations: MUGA: multi-gated acquisition; HsTnT: high-sensitivity cardiac troponin T; CEpOP: cyclophosphamide, epirubicin, vincristine, and prednisone or cyclophosphamide; CHOP: cyclophosphamide, doxorubicin, vincristine, and prednisone; R: rituximab; and LVEF: left ventricular ejection fraction.

**Figure 4:**
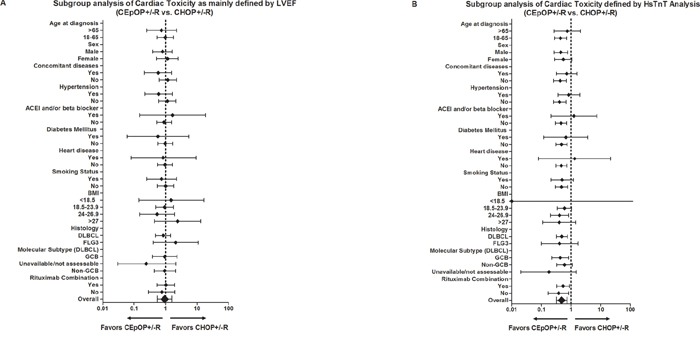
Hazard ratio risk analysis for cardiac toxicity (changes in LVEF A. or HsTnT levels B.) development in patients treated with CEpOP+/−R or CHOP+/−R Abbreviations: LVEF: left ventricular ejection fraction; HsTnT: high-sensitivity cardiac troponin T; CEpOP: cyclophosphamide epirubicin, vincristine, and prednisone or cyclophosphamide; CHOP: cyclophosphamide, doxorubicin, vincristine, and prednisone; R: rituximab; ACEI: angiotensin converting enzyme inhibitors; BMI: body mass index; DLBCL: diffuse large B-cell lymphoma; FLG3: follicular lymphoma grade 3; GCB: germinal center B-cell.

A total of 348 patients completed all LVEF evaluations during the study period. An objective decrease in LVEF (≥ 10%) from baseline was observed in 56 patients. Two of these patients (CEpOP+/−R=1, CHOP+/−R=1) developed symptoms consistent with clinical heart failure and did not complete more than 4 cycles of their planed treatment. In the remaining 54 patients, there was no difference in the incidence of decreased LVEF between treatment groups (CEpOP+/−R=27/180, 15.0% vs. CHOP+/−R=27/168, 16.1%, *P* = 0.783), and these patients did not develop symptoms of heart failure during the study. The addition of rituximab to systemic chemotherapy did not increase the incidence of LVEF reduction (Figure 3A-3C, 4A, Supplementary Table 1). Furthermore, the risk of reduced LVEF between patients treated with CHOP+/−R versus CEpOP+/−R was similar even after adjustments for age, sex, co-morbidities (hypertension, diabetes mellitus, and history of heart disease), smoking status, BMI, histology subtype, molecular subtype of DLBCL, and use of rituximab (Figure 4A). Follow-ups will be done to observe long-term cardiotoxicity in these patients with decreased LVEF.

### Elevated serum HsTnT levels in DLBCL/FLG3 patients treated with CHOP+/−R or CEpOP+/−R

In the 348 patients who completed all LVEF evaluations, serum HsTnT was measured in 324 patients. Prior to therapy, 8 patients exhibited elevated HsTnT, and their distribution was similar in the CHOP+/−R and CEpOP+/−R groups (2/167, 1.2% vs. 6/157, 3.8%, *P* = 0.128, Supplementary Table 2). Following the completion of 4 cycles of therapy, 100 patients were found to have elevated HsTnT levels; all were asymptomatic and clinically and hemodynamically stable. More patients in the CHOP+/−R treated (66/157) group exhibited elevated HsTnT after 4 cycles of therapy than in the CEpOP+/−R treated group (34/167) (42.0% vs. 20.4%, *P* = 0.001) (Figure 3D-3F, 4B). Subgroup analysis identified several subgroups of patients treated with CEpOP+/−R that had a lower risk of elevated HsTnT levels compared to those treated with CHOP+/−R: those between 18-65 years old (0.44, 95% CI: 0.28-0.70), males (0.45, 95% CI: 0.27-0.77), those without comorbid conditions (0.43, 95% CI: 0.26-0.69), normotensive patients (0.41, 95% CI: 0.26-0.67), patients who received angiotensin converting enzyme (ACE) inhibitors and/or beta-blockers during antineoplastic therapy (0.46, 95% CI: 0.30-0.70), non-diabetic patients (0.48, 95% CI: 0.31-0.73), those without a prior history of heart disease (0.47, 95% CI: 0.31-0.72), non-smokers (0.48, 95% CI: 0.30-0.77), those with BMIs between 24-26.9 (0.41, 95% CI: 0.20-0.84), DLBCL patients (0.49, 95% CI: 0.32-0.76), molecular subtype GCB patients (0.43, 95% CI: 0.22-0.83), and those who received rituximab combination treatment (0.54, 95% CI: 0.33-0.88) (Figure 4B).

### Risk factor analysis of patients with elevated HsTnT after 4 cycles of CEpOP+/−R or CHOP+/−R

Patients who had higher baseline HsTnT levels were more likely to have elevated HsTnT levels after 4 cycles of CEpOP+/−R or CHOP+/−R (*P* = 0.001, Table 3). Patients who had elevated HsTnT after 4 cycles of CEpOP+/−R or CHOP+/−R had higher levels of HsTnT and ΔHsTnT compared to their normal counterparts (*P* < 0.001 for both).

**Table 3:** Risk factor analysis of patients with elevated HsTnT after 4 cycles of CEpOP+/−R or CHOP+/−R

	Patients with post-4 cycles of HsTnT normal, No. (%)	Patients with post-4 cycles of HsTnT elevated, No. (%)	*P* Value
No. of patients	224	100	
Baseline HsTnT levels (Mean ± Std, ug/L)	0.004 ± 0.002	0.007 ± 0.010	0.001
Post-4 cycles of HsTnT levels (Mean ± Std, ug/L)	0.007 ± 0.003	0.021 ± 0.011	0.000
ΔHsTnT (Mean ± Std, ug/L)	0.003 ± 0.003	0.014 ± 0.013	0.000
Baseline positive HsTnT	1 (0.4)	7 (7.0)	0.000
Post-4 cycles of HsTnT (Subgroups)			
Age at diagnosis, years			0.002
>65	11 (4.9)	15 (15.0)	
18-65	213 (95.1)	85 (85.0)	
Sex			0.017
Male	109 (48.7)	63 (63.0)	
Female	115 (51.3)	37 (37.0)	
Concomitant diseases			0.090
Yes	36 (16.1)	24 (24.0)	
No	188 (83.9)	76 (76.0)	
Hypertension			0.064
Yes	29 (12.9)	21 (21.0)	
No	195 (87.1)	79 (79.0)	
ACEI and/or beta-blocker			0.287
Yes	6 (2.7)	5 (5.0)	
No	218 (97.3)	95 (95.0)	
Diabetes mellitus			0.321
Yes	8 (3.6)	6 (6.0)	
No	216 (96.4)	94 (94.0)	
Heart disease			0.894
Yes	5 (2.2)	2 (2.0)	
No	219 (97.8)	98 (98.0)	
Smoking status			0.743
Yes	53 (23.7)	22 (22.0)	
No	171 (76.3)	78 (78.0)	
BMI, kg/m^2^			0.122
<18.5	11 (4.9)	2 (2.0)	
18.5-23.9	136 (60.7)	53 (53.0)	
24-26.9	62 (27.7)	32 (32.0)	
>27	15 (6.7)	13 (13.0)	
Histology			0.885
DLBCL	205 (91.5)	92 (92.0)	
FLG3	19 (8.5)	8 (8.0)	
Molecular subtype (DLBCL) / Number of DLBCL			0.057
GCB	61/205 (29.8)	39/92 (42.4)	
Non-GCB	118/205 (57.6)	47/92 (51.1)	
Unavailable/not assessable	26/205 (12.7)	6/92 (6.5)	
Rituximab combination			0.922
Yes	158 (70.5)	70 (70.0)	
No	66 (29.5)	30 (30.0)	

Note: ΔHsTnT: The difference between baseline and post-4 cycles of treatment on HsTnT levels; Normal HsTnT is 0-0.014ug/L.

Abbreviation: HsTnT = high sensitive serum cardiac troponin T; CEpOP+/−R = cyclophosphamide, epirubicin, vincristine and prednisone +/− rituximab; CHOP+/−R = cyclophosphamide, doxorubicin, vincristine, and prednisone +/− rituximab; LVEF = left ventricular eject fraction; ACEI = angiotensin converting enzyme inhibitors; BMI = body mass index; DLBCL = diffuse large B cell lymphoma; FLG3 = follicular lymphoma grade 3; GCB = germinal center B-cell-like.

The proportion of older patients and males was higher among those with elevated HsTnT after 4 cycles of CEpOP+/−R or CHOP+/−R than among those with normal HsTnT after 4 cycles of treatment (age > 65y: 15.0% vs. 4.9%, *P* = 0.002; male: 63.0% vs. 48.7%, *P* = 0.017)

## DISCUSSION

In the pre-rituximab era, randomized phase III clinical studies comparing anthracycline-based regimens in cancer patients aimed to determine differences in anti-tumor activity and safety (including the incidence of cardiac toxicity) among anthracycline agents (doxorubicin, idarubicin, or epirubicin) or analogs (i.e. mitoxantrone). Mitoxantrone-based front-line chemotherapy was found to be inferior to doxorubicin-based chemotherapy in elderly DLBCL patients. CHOP therapy also resulted in better CR rates, PFS, and OS than CNOP therapy (cyclophosphamide, mitoxantrone, vincristine and prednisolone) in these patients. CHOP treatment increased lymphoma-specific survival three years after treatment (42%) compared to CNOP treatment (26%) (*P* = 0.034). The risk of cardiac toxicity was similar between these two regimens [22]. In a smaller clinical study (N = 211), Nair *et al.* found that the efficacy and safety of an epirubicin-based regimen (cumulative dose 480mg/m^2^) and a doxorubicin-based regimen (cumulative dose 300mg/m^2^) were equivalent in previously untreated DLBCL patients [23].

Here, we compared two anthracycline-based regimens with or without rituximab and demonstrated that epirubicin (70 mg/m^2^) was equivalent to doxorubicin (50 mg/m^2^) in terms of anti-tumor activity. Additionally, the risk of decreased LVEF after 4 cycles of cheomtherapy did not differ between patients treated with CEpOP+/−R and those treated with CHOP+/−R.

Novel biomarkers that might predict heart injury during chemotherapy before changes in LVEF are evident have recently been identified [24, 25]. Serum troponins are sensitive and specific biomarkers for evaluating ischemic heart damage and/or myocardial infarction in the clinical setting [26]. Early elevated serum TnT during anthracycline treatment was associated with increased long-term anthracycline-induced cardiotoxicity [18]. Here, we found that serum TnT levels changed during therapy. Interestingly, elevated HsTnT was more common among patients who received CHOP+/−R treatment that among those receiving CEpOP treatment. Long-term follow up is needed to determine whether these elevated HsTnT levels will translate into differences in the incidence of cardiac toxicity over time between treatment groups.

The development and validation of rituximab non-anthracycline-based regimen(s) in aggressive B-cell lymphoma might provide an effective alternative for patients with a higher risk of developing anthracycline-induced cardiotoxicity. In a Canadian study, patients with a contraindication to anthracycline-based therapy received rituximab in combination with cyclophosphamide, etoposide, vincristine, and prednisone (R+CEOP) every 21 days for 6 cycles. The 5-year PFS rate was similar in R-CEOP treated patients and R-CHOP historical controls [27].

In conclusion, the CEpOP+/−R treatment regimen has similar short-term efficacy and is associated with a lower incidence of febrile neutropenia compared to the CHOP+/−R regimen. While no differences in incidence of decreased LVEF were observed between these two groups, CEpOP+/−R increased HsTnT levels less often than CHOP+/−R. Longer follow-ups are needed to monitor the risk of cardiac dysfunction and to determine whether differences in elevated HsTnT after epirubicin or doxorubicin therapy justify changes in clinical practices.

## PATIENTS AND METHODS

### Patients

The study has been described in detail previously [19]. Briefly, the main inclusion criteria were as follows: previously untreated and histologically confirmed DLBCL or FLG3 according to the 2008 World Health Organization (WHO) classification; age ≥ 18 and ≤ 75 years; Eastern Cooperative Oncology Group (ECOG) performance status (PS) index ≤ 2; normal cardiac function (LVEF ≥ 50% by MUGA); and adequate bone marrow (ANC ≥ 1.5×10^9^/L, PLT ≥ 100×10^9^/L, Hb ≥ 80g/L), hepatic (total bilirubin, ALT and AST were ≤ 1.5 × upper limit of normal [UNL]), and renal (Cr < 1.5×UNL and creatinine clearance ≥ 50ml/min) function.

The main cardiac-related exclusion criteria were as follows: prior history of myocarditis, myocardial ischemia, myocardial infarction, arrhythmia requiring medical intervention, and clinical or subclinical pericardial effusion. Patients were also excluded if they had any of the following: a prior history of other cancers, except treated cervical or basal cell skin carcinoma; organ transplantation; severe active infection (i.e. hepatitis, syphilis, or human immunodeficiency virus [HIV] infection); lymphoma with documented central nervous system or leptomeningeal involvement; and severe neurological or psychiatric conditions. Pregnant or lactating women were also excluded.

Complete medical histories were collected for all patients and included documentation of any prior cardiovascular illness (i.e. hypertension, angina, coronary artery disease), prior metabolic conditions (i.e. diabetes, hyperlipidemia, etc.), other medical conditions (i.e. chronic obstructive lung disease [COPD]), relevant social history (i.e. smoking history), and a complete physical exam (including measurement of body mass index [BMI] and determination of ECOG-PS index). In addition, patients underwent imaging studies consisting of a contrast-enhanced computed tomography scan (CT-scan) of the neck, thorax, abdomen, and pelvis, and collection of bone marrow biopsies, for staging purposes. Baseline electrocardiograms (ECG) and MUGA scans were performed to assess baseline heart rhythm and LVEF, respectively, and laboratory tests to document liver, kidney, and bone marrow function were performed.

### Treatment

The clinical study was conducted in accordance with the Declaration of Helsinki. All patients received and signed informed consent. The clinical trial received ethical approval from the Research Ethics Committee at the Fudan University Shanghai Cancer Center (FUSCC). A stratified randomization schedule was generated by age (greater or less than 65) using SAS 9.3 (SAS Institute Inc., Cary, NC, USA). Eligible patients were randomized at a ratio of 1:1 to receive cyclophosphamide at 750mg/m^2^ on day D1, vincristine at 1.4mg/m^2^ on D1 (capped at 2mg/dose), 100mg prednisone on D1-5, and either epirubicin at 70mg/m^2^ on D1 (CEpOP) or doxorubicin at 50mg/m^2^ on D1 (CHOP) with or without rituximab (based on antibody availability) at 375mg/m^2^ on day 1 of each CHOP or CEpOP cycle. These cycles were repeated every 21 days for a total of 6-8 cycles. Radiotherapy for any residual tumor(s) with active disease as determined by post-therapy imaging studies or for original sites of disease bulk (> 7.5 cm^2^) after completion of chemotherapy +/− rituximab was permitted at discretion of the clinical investigator. Patients considered high risk for CNS relapse received 12mg intrathecal methotrexate once a week for a total of four doses in the first month of therapy.

Growth factor support was not mandated for all patients but was administered if needed. Interleukin 11 (IL-11) and dexrazoxane were not allowed. Adverse events assessment and severity of toxicities were performed at each clinic visit according to the National Cancer Institute Common Terminology Criteria (CTC) for Adverse Events version 3.0. Dose reductions were advised in patients experiencing grade 4 treatment-related toxicities and were as follows: epirubicin/doxorubicin and cyclophosphamide were reduced by 20% in patients developing CTC grade 4 thrombocytopenia, neutropenia or febrile neutropenia; epirubicin/doxorubicin and vincristine were reduced by 20% in patients developing grade 3/4 liver dysfunction; and vincristine was reduced by 20% if grade 3 or 4 peripheral neurotoxicity was observed. A maximum of two dose reductions were allowed for each patient.

### Efficacy and adverse events assessment

Efficacy evaluation was performed after every two cycles of treatment and consisted of history and physical examination, laboratory work, toxicity assessment, and imaging studies. End of therapy procedures included history and physical examination, toxicity assessment, and imaging studies. Bone marrow biopsy at the end of therapy was repeated only in those patients with baseline bone marrow involvement with lymphoma. Response was assessed in accordance to the International Workshop on Standardized Response Criteria for NHL [20]. In the follow up period, patients were evaluated at 6 and 12 months after the completion of treatment and underwent routine history and physical examinations, laboratory work, and imaging studies.

### LVEF by MUGA scan and biomarker assays

LVEF assessments were performed at baseline, after the completion of 4 cycles of either CHOP+/−R or CEpOP+/−R, and subsequently at the discretion of the treating physician by MUGA scan (GE infinia; Software version: Xeleris 3.0423). We chose this time frame to evaluate cardiotoxicity as early as possible in order to have the option of switching to a non-anthracycline regimen. All MUGA scan studies were analyzed by the same cardiologist (BZ). In addition, serum samples were collected for TnT measurements at the time of enrollment and after 4 cycles of therapy. Samples were sent to the FUSCC central laboratory, frozen, and stored at −80°C until analysis was performed. Hemolyzed or insufficient samples were excluded. Investigators performing serum assays remained blinded throughout the study. Serum TnT concentrations were measured using the high-sensitivity cardiac TnT (HsTnT) one-step electrochemiluminescence immunoassay (Roche cobase 411) as previously described [21]. The sensitivity and specificity of the assay were 99% and 98%, respectively. The threshold for an abnormally elevated value using this method is 0.014μg/L, and the range of detection extends from 0.003 to 1000 μg/L [21].

### Primary and secondary endpoints

The primary endpoint was the incidence of cardiac events, defined as LVEF < 50% during or after treatment, a decrease from baseline LVEF during treatment of at least 10%, or clinical heart failure with New York Heart Association Functional Class ≥ 2 [8]. Secondary endpoints included treatment-related toxicities, ORR, complete remission (CR) rates, partial remission (PR) rates, PFS, and OS in patients treated with CHOP+/−R vs. CEpOP+/−R. A post-hoc analysis was performed to determine the association between the type of therapeutic approach and HsTnT levels and incidence of cardiac events during or after treatment.

### Statistical analysis

We calculated that 320 participants (160 per treatment) would have 90% power to detect an 11% decrease in primary endpoint incidence after epirubicin treatment, compared to 20% incidence in the control group, after 4 cycles of treatment at a two-sided type I error rate of 0.05. To account for drop-outs, 400 patients needed to be enrolled for the study. All analyses were performed on an intention-to-treat basis. Continuous variables are expressed as means and standard deviations or medians and quartile ranges. Categorical variables are expressed as frequencies. Student's *t*-tests, Wilcoxon signed rank tests, Chi-square tests, or Fisher's exact tests were applied to detect differences between the treatment groups. PFS was compared using the Kaplan-Meier method and log-rank test. The Cox proportional hazards model was used to estimate hazard ratios and associated 95% confidence intervals. All analyses were conducted using SPSS Version 13.0 (Chicago, IL); all graphs were made using GraphPad Prism Version 5.0 (GraphPad Software, Inc.).

## SUPPLEMENTARY TABLES




