# Supplementary material for: Cardiotoxicity as indicated by LVEF and troponin T sensitivity following two anthracycline-based regimens in lymphoma: Results from a randomized prospective clinical trial
Source: Oncotarget. 2016 Apr 11;7(22):32519–31. doi: 10.18632/oncotarget.8685 (PMC5078030; doi:10.18632/oncotarget.8685)
Supplement: Supplementary file 1 [file oncotarget-07-32519-s001.pdf]

Cardiotoxicity as indicated by LVEF and troponin T sensitivity following two anthracycline-based regimens in lymphoma: Results from a randomized prospective clinical trial

SUPPLEMENTARY TABLES

Supplementary File 1: Comparison Study of Doxorubicin Versus Epirubicin-induced Cardiac toxicity in Patients with DLBCL/FLG3

See Supplementary File 1.

Supplementary Table S1: Subgroup analysis of cardiac toxicity defined mainly by LVEF

	CEpOP+/-R	CHOP+/-R	*P* value
No. of patients	180	168	
No. with decreased LVEF who suffered from cardiac toxicity (mean ± Std %)	11.9 ± 2.5	12.4 ± 2.4	0.610
No. with a decreased LVEF (%)	28 (15.6)	28 (16.7)	0.778
			P value for interaction
Age at diagnosis (years)			<0.001
>65	6/15 (40.0)	7/13 (53.8)	
18-65	22/165 (13.3)	21/155 (13.5)	
Sex			0.972
Male	14/93 (15.1)	17/90 (18.9)	
Female	14/87 (16.1)	11/78 (14.1)	
Concomitant diseases			0.491
Yes	6/32 (18.8)	11/34 (32.4)	
No	22/148 (14.9)	17/134 (12.9)	
Hypertension			0.228
Yes	6/27 (22.2)	10/27 (37.0)	
No	22/153 (14.4)	18/141 (12.8)	
ACEI and/or beta blocker			0.949
Yes	2/6 (33.3)	1/5 (20.0)	
No	26/174 (14.9)	27/163 (16.6)	
Diabetes mellitus			0.851
Yes	1/7 (14.3)	3/12 (25.0)	
No	27/173 (15.6)	25/156 (16.0)	
Heart disease			0.302
Yes	1/3 (33.3)	2/5 (40.0)	
No	27/177 (15.3)	26/163 (16.0)	
Smoking status			0.733
Yes	6/43 (14.0)	7/37 (18.9)	
No	22/137 (16.1)	21/131 (16.0)	
BMI, kg/m^2			0.974
<18.5	2/8 (25.0)	1/6 (16.7)	
18.5-23.9	18/99 (18.2)	17/94 (18.1)	
24-26.9	4/57 (7.0)	6/46 (13.0)	
>27	4/15 (26.7)	2/17 (11.8)	
Histology			0.892
DLBCL	23/162 (14.2)	26/153 (17.0)	
FLG3	5/18 (27.8)	2/15 (13.3)	
Molecular subtype (DLBCL)			0.913
GCB	10/57 (17.5)	9/48 (18.8)	
Non-GCB	12/87 (13.8)	13/88 (14.8)	
Unavailable/not assessable	1/18 (5.6)	4/17 (23.5)	
Rituximab combination			0.723
Yes	20/126 (15.9)	19/122 (15.6)	
No	8/54 (14.8)	9/46 (19.6)	

Abbreviations: LVEF = left ventricular eject fraction; CEpOP+/-R = cyclophosphamide, epirubicin, vincristine and prednisone +/- rituximab; CHOP+/-R = cyclophosphamide, doxorubicin, vincristine, and prednisone +/- rituximab; ACEI = angiotensin converting enzyme inhibitor; BMI = body mass index; DLBCL = diffuse large B-cell lymphoma; FLG3 = follicular lymphoma grade 3; GCB = germinal center B-cell-like.

Supplementary Table S2: Subgroup analysis of cardiac toxicity as defined by elevated HsTnT levels

	CEpOP+/-R	CHOP+/-R	*P* value
No. of patients	167	157	
Baseline HsTnT levels (Mean ± Std, ug/L)	0.004 ± 0.003	0.005 ± 0.008	0.192
HsTnT levels after 4 treatment cycles (Mean ± Std, ug/L)	0.009 ± 0.006	0.014 ± 0.011	< 0.001
ΔHsTnT (Mean ± Std, ug/L)	0.005 ± 0.005	0.008 ± 0.012	< 0.001
No. of patients with elevated baseline HsTnT levels (%)	2 (1.2)	6 (3.8)	0.128
No. of patients with elevated HsTnT levels after 4 treatment cycles (%)	34 (20.4)	66 (42.0)	< 0.001
After 4 treatment cycles (Subgroups)			*P* value for interaction
Age at diagnosis (years)			0.090
>65	7/14 (50.0)	8/12 (66.7)	
18-65	27/153 (17.6)	58/145 (40.0)	
Sex			0.096
Male	20/87 (23.0)	43/85 (50.6)	
Female	14/80 (17.5)	23/72 (31.9)	
Concomitant diseases			0.530
Yes	10/30 (33.3)	14/30 (46.7)	
No	24/137 (17.5)	52/127 (40.9)	
Hypertension			0.401
Yes	10/26 (38.5)	11/24 (45.8)	
No	24/141 (17.0)	55/133 (41.4)	
ACEI and/or beta-blocker			0.900
Yes	3/6 (50.0)	2/5 (40.0)	
No	31/161 (19.3)	64/152 (42.1)	
Diabetes mellitus			0.370
Yes	2/6 (33.3)	4/8 (50.0)	
No	32/161 (19.9)	62/149 (41.6)	
Heart disease			0.861
Yes	1/3 (33.3)	1/4 (25.0)	
No	33/164 (20.1)	65/153 (42.5)	
Smoking status			0.557
Yes	8/40 (20.0)	14/35 (40.0)	
No	26/127 (20.5)	52/122 (42.6)	
BMI, kg/m^2			0.321
<18.5	0/7 (0.0)	2/6 (33.3)	
18.5-23.9	20/95 (21.1)	33/94 (35.1)	
24-26.9	11/53 (20.8)	21/41 (51.2)	
>27	3/12 (25.0)	10/16 (62.5)	
Histology			0.518
DLBCL	31/151 (20.5)	61/146 (41.8)	
FLG3	3/16 (18.8)	5/11 (45.5)	
Molecular subtype (DLBCL)			0.268
GCB	13/54 (24.1)	26/46 (56.5)	
Non-GCB	17/80 (21.3)	30/85 (35.3)	
Unavailable/not assessable	1/17 (5.9)	5/15 (33.3)	
Rituximab Combination			0.853
Yes	25/116 (21.6)	45/112 (40.2)	
No	9/51 (17.6)	21/45 (46.7)	

Note: ΔHsTnT: The difference between HsTnT levels at baseline and after 4 treatment cycles; Normal HsTnT is 0-0.014ug/L; All subgroups analyses are after 4 treatment cycles.

Abbreviation: HsTnT, high-sensitivity serum cardiac troponin T; CEpOP+/-R = cyclophosphamide, epirubicin, vincristine and prednisone +/- rituximab; CHOP+/-R = cyclophosphamide, doxorubicin, vincristine, and prednisone +/- rituximab; ACEI = angiotensin converting enzyme inhibitors; BMI = body mass index; DLBCL = diffuse large B-cell lymphoma; FLG3 = follicular lymphoma grade 3; GCB = germinal center B-cell-like.
