# Supplementary material for: Cardiotoxicity as indicated by LVEF and troponin T sensitivity following two anthracycline-based regimens in lymphoma: Results from a randomized prospective clinical trial
Source: Oncotarget. 2016 Apr 11;7(22):32519–31. doi: 10.18632/oncotarget.8685 (PMC5078030; doi:10.18632/oncotarget.8685)
Supplement: Supplementary file 2 [file oncotarget-07-32519-s002.docx]

Comparison Study of Doxorubicin Versus Epirubicin-induced Cardiac toxicity in Patients with DLBCL/FLG3

Lymphoma Multidisciplinary Study Group, LMSG

Fudan University Shanghai Cancer Center

Shanghai 200032, China

**Version: 1.3**

1. Background

Diffuse large B cell lymphoma (diffuse large B-cell lymphoma, DLBCL) is the most common subtype of non-Hodgkin’s lymphoma (NHL), accounting for about 25% of NHL. The proportion in Asian countries may be higher [1]. According to WHO classification, DLBCL is an aggressive NHL, and more than 60% of these patients are diagnosed in stage III/IV. Follicular lymphoma (FL) is the second most common NHL subtype, accounting for about 20%, and is classified as indolent NHL. Based on the number of centroblastic cells under high magnification, FL can be divided into grade 1 (0-5), grade 2 (6-15), and grade 3 (more than 15); grade 1 is the most common. The biological behavior of grade 3 FL tumors is similar to DLBCL tumors, so the two are often treated similarly [2].

CHOP (cyclophosphamide, doxorubicin, vincristine and prednisone) has become the standard chemotherapy regimen for aggressive NHL, especially DLBCL. In several randomized phase III trials [3], the CHOP regimen was the most effective. In recent years, the superiority of R-CHOP over CHOP has been demonstrated in both elderly and younger patients in terms of tumor remission rate and overall survival [4, 5].

Eight cycles of this chemotherapy regimen are performed in most studies, although the RICOVER-60 study showed that there was no difference between 6 cycles and 8 cycles in terms of R-CHOP-14. Whether the more common R-CHOP-21 regimen is equally effective after 8 cycles or 6 cycles of chemotherapy remains unknown [6]. According to the guidelines of the NCCN (Year 2008 Version 3), 6-8 cycles are optional. Treatment of stage I/II DLBCL differs greatly between the United States and Europe. In the United States, patients without a large mass (diameter ≥ 10cm) usually receive 3-4 cycles of chemotherapy followed by local radiotherapy treatment. In Europe, chemotherapy alone is the main treatment method, and radiotherapy is only used for those with large masses or who fail to achieve a complete remission. Many patients who receive CHOP/R-CHOP treatment achieve long-term survival, and long-term toxicity problems, particularly cardiac toxicity caused by doxorubicin, caused by chemotherapy have become more common. Doxorubicin is the most commonly used anthracycline, and its anti-tumor effects are mainly due to its interference with DNA, RNA and protein synthesis. Cardiac toxicity, which limits the usable dose of doxorubicin, mainly results from the effects of oxygen free radicals induced by lipid peroxidation, which often results in irreversible myocardial cell vacuolation, damage, and replacement by fibrous tissue [7]. Cardiotoxicity induced by doxorubicin can be divided into 3 categories based on clinical manifestation: acute, early, and late [8]. Acute toxicity, which is often rare, self-limited, and requires no special treatment, occurs approximately 1 week after treatment, and mainly presents as abnormal ECG and myocardial systolic function. Early toxicity occurs either during or within one year of treatment and is mainly due to damage to myocardial cells, which leads to a serious decline in myocardial systolic function or even congestive heart failure (CHF). Late toxicity occurs more than one year after treatment and often leads to heart failure; this toxicity is especially common in patientsPediatric patients whose tumor cure rate is relatively high.

Many studies show that the cumulative anthracycline dose is the most important risk factor for cardiac toxicity. In an early study, when patients received accumulated doxorubicin doses of 400mg/m^2^, 550mg/m^2^_,_ and 700mg/m^2^, the incidences of CHF were 3%, 7% and 18% respectively; thus, 550mg/m^2^ became the clinical dose limit for doxorubicin accumulation [9]. However, recent studies show that CHF incidence is probably greatly underestimated. Swain *et* *al.* analyzed 630 patients who received doxorubicin in a phase III randomized trial; the cumulative doses were 400mg/m^2^, 500mg/m^2^_,_ and 550mg/m^2^, and the incidences of CHF were 5%, 16% and 26%, respectively. Once the cumulative dose reached more than 650mg/m^2^, the incidence of CHF was as high as 48% [10]. In order to reduce the cardiac toxicity of doxorubicin, a novel preparation (liposomal doxorubicin) has been produced. Although the heart toxicity is significantly reduced, its high price limits its use in clinical practice, and whether it can completely replace doxorubicin still requires investigation in randomized controlled phase III studies. In addition, dexrazoxane has been approved by the US FDA as a cardiac protective agent, but it is mainly for patients receiving accumulated doses of doxorubicin more than 300mg/m^2^, and the benefits of its early use are not clear; in addition, the drug might have tumor-protective effects.

Although controlling the cumulative dose of doxorubicin can reduce the risk of cardiac toxicity, some patients may still suffer from CHF at lower cumulative doses. Therefore, there is an urgent need to effectively distinguish the population of patients with a high cardiotoxicity risk and discontinue anthracycline treatment in these patients at an appropriate time. Of the available monitoring methods, left ventricular ejection fraction (LVEF) detected by radionuclide angiography (RNA) is the most clinically established and widely used. Schwartz *et* *al.* found that LVEF correlates well with CHF and developed a guideline for LVEF monitoring of cardiac toxicity during doxorubicin treatment. [11] A previous study showed that only 2 out of 70 high-risk patients (3%) had heart failure when this guideline was followed; on the contrary, 44 out of 212 high-risk patients (21%) had heart failure when it was not followed. In addition to LVEF, echocardiography is another commonly used monitoring method. However, compared to RNA, this method has poor reproducibility and objectivity, and a high technical level operator is required; it is therefore mainly used in pediatric oncology at present. Mousiainen *et* *al.* compared the two methods and obtained inconsistent results; they concluded that the guideline for RNA monitoring cardiac toxicity does not apply to echocardiography, and RNA is still the standard method [12]

In addition to LVEF, researchers have attempted to identify serum biomarkers for predicting or detecting early cardiac toxicity, and most current research focuses mainly on troponin and natriuretic peptides. At present, cardiac troponin has diagnostic and prognostic value for predicting myocardial ischemia [13]. In a phase III randomized trial, 206 patients with acute lymphoblastic leukemia (ALL) received either doxorubicin monotherapy or doxorubicin combined with dexrazoxane (a cardio-protective agent) [14]. There was no difference between the two groups after treatment in LVEF detected by echocardiography. The percentage of patients with elevated cardiac troponin T (troponin T) in the doxorubicin alone group was higher than that in the combination group (50% vs. 21%, P<0.001), suggesting that troponin T may be more sensitive than LVEF in monitoring cardiac toxicity. In addition, both troponin T and troponin have been shown to correlate with long-term decreased cardiac systolic and diastolic function [15-18]. Natriuretic peptide mainly consists of atrial natriuretic peptide (ANP) and brain natriuretic peptide (BNP), produced by an atrium and ventricle respectively, and it enhances myocardial function in cardiac load conditions. In patients with heart failure, elevated BNP assists in early diagnosis and predicting prognosis [19]. Similarly, in doxorubicin studies, ANP and BNP were found to be associated with systolic and diastolic function [20-21]. Although these biomarkers show diagnostic and prognostic value, generally accepted threshold levels have not been established. In addition, other factors can easily interfere with detection objectivity, and these markers cannot yet replace the monitoring role of LVEF.

In addition to doxorubicin, epirubicin is another commonly used anthracycline antibioticsin in the treatment of breast cancer and soft tissue sarcoma. Compared with doxorubicin, epirubicin has higher dose of cumulative toxicity; the incidences of CHF were 4.3% and 15% at 900mg/m^2^ and 1000mg/m^2^_,_ respectively [22]. Launchbury *et* *al.* analyzed several random trials comparing doxorubicin and epirubicin in the treatment of breast cancer and found that these two drugs had equivalent effects, but the same dose of epirubicin was associated with lower incidence of hematologic, non-hematologic and cardiac toxicity compared to doxorubicin [23]. Nair *et* *al.* compared treatment outcomes of the MACOP-B regimen (50mg/m^2^ doxorubicin) and the MACOP-B regimen (75mg/m^2^ epirubicin) in NHL [24]. The results demonstrated that there was no significant difference between these two anthracycline regimens in terms of efficacy and toxicity. In recent years, many studies have used epirubicin instead of doxorubicin in the CHOP regimen and shown satisfactory therapeutic effects [25-27]. The optimal dose of epirubicin has not been established, but two recent randomized trials and our center used 70mg/m^2^ [28, 29]. Compared to doxorubicin, epirubicin may be administered at a higher cumulative dose with less cardiac toxicity. Based on the above research, our lymphoma multidisciplinary study group plans to conduct a randomized phase III study comparing CHOP/R-CHOP- and CEPOP/R-CEPOP-related cardiac toxicity in the treatment of aggressive B cell non Hodgkin lymphoma.

**2. Study design**

A single center, open label, phase III randomized controlled clinical study, plans to enroll 320 patients who randomly receive CEpOP+/-R regimen or CHOP+/-R regimen at a 1:1 ratio.

**3. Endpoint**

3.1 Primary endpoints

Incidence of cardiac events:

Clinical: heart failure according to New York Heart Association Functional

Class >2.

Subclinical: LVEF < 50% during or post-treatment, a decrease in LVEF > 10% during treatment

3.2 Secondary endpoints

Overall response rate (ORR): complete remission (CR) rates, partial remission (PR) rates

Overall survival (OS): the interval between the date of randomization and death from any cause.

Progression free survival (PFS): the interval between the date of randomization and the occurrence of one the following events: disease progression, relapse and death from any cause.

Remission duration: the interval from CR/CRu/PR to disease recurrence or progression

Adverse events: hematological and non-hematological toxicity (NCI CTCAE v3.0）

**4. Inclusion criteria and exclusive criteria**

4.1 Inclusion criterias:

1) Ages: 18-75 years old

2) Previously untreated and histologically confirmed DLBCL or FLG3 according to the 2008 World Health Organization (WHO) classification, scheduled to receive at least 6 cycles of chemotherapy treatment

3) Eastern Cooperative Oncology Group (ECOG) performance status (PS) index < 2

4) Informed consent available

5) Life expectancy of more than 3 months;

6) Cardiac function: RNA detection of LVEF ≥ 50% and EKG without myocardial ischemia;

7) Bone marrow function: ANC ≥ 1.5 × 10^9^/L, PLT ≥ 100 × 10^9^/L, Hb ≥ 80 g/L;

8) Liver function: total bilirubin, ALT and AST were <1.5 × UNL (the upper limit of normal value)

9) Renal function: Cr<1.5 × UNL and creatinine clearance > 50ml/min

4.2 Exclusion criterias:

1) Prior history of myocarditis, myocardial ischemia, myocardial infarction, arrhythmia requiring medical intervention

2) Significant pericardial effusion showed by chest CT scan

3) Prior history of other cancers except treated cervical or basal cell skin carcinoma, organ transplantation

4) Syphilis or human immunodeficiency virus (HIV) infection

5) Pregnant or lactating women

6) History of organ transplantation

7) Serious active infections (including hepatitis)

8) Serious neurological or psychiatric history, including dementia or epilepsy.

4.3 Termination criteria:

1) Withdrew consent

2) Researchers think it is necessary to terminate the study;

3) Disease progression or death;

4) Poor compliance

5) Subclinical or clinical cardiac toxicity;

6) Unable to continue treatment because of severe toxicity

**5 Regimens**

5.1 Experimental arm: CEpOP or R-CEpOP, each course of treatment every 3 weeks, at least 6 courses

CEpOP:

Cyclophosphamide 750mg/m^2^ IV D1 (Endoxan, Baxter)

Epirubicin 70mg/m^2^ IV D1 (Pharmorubcin, Pfizer Inc)

Vincristine 1.4mg/m^2^ IV D1 (2 mg)

Prednisone 50mg BID PO D1~5

R-CEpOP:

Rituximab, 375 mg/m^2^ IV D0 (trade name: rituximab, Roche)

Cyclophosphamide 750 mg/m^2^ IV D1 (Endoxan, Baxter)

Epirubicin 70 mg/m^2^ IV D1 (Pharmorubcin, Pfizer Inc)

Vincristine 1.4 mg/m^2^ IV D1 (2 mg)

Prednisone 50 mg BID PO D1~5

5.2 control group: CHOP or R-CHOP, each course of treatment every 3 weeks, at least 6 courses

CHOP:

Cyclophosphamide 750 mg/m^2^ IV D1 (Endoxan, Baxter)

Doxorubicin and 50 mg/m^2^ IV D1 (Doxorubicin hydrochloride, Pfizer Inc)

Vincristine, 1.4 mg/m^2^ IV D1 (2mg)

Prednisone 50 mg BID IV D1~5

R-CHOP：

Rituximab 375 mg/m^2^ IV D0 (Rituximab, Roche)

Cyclophosphamide 750 mg/m^2^ IV D1 (Endoxan, Baxter)

Doxorubicin 50 mg/m^2^ IV D1 (Doxorubicin hydrochloride, Pfizer Inc)

Vincristine, 1.4 mg/m^2^ IV D1 (2mg)

Prednisone 50 mg BID PO D1~5

5.3 Supplement

1) All patients receive at least 6 courses of chemotherapy, and 2 cycles of consolidation are recommended after achieving the best therapeutic outcome.

2) Dosage is strictly calculated according to body surface area during the first cycle of treatment dosage; if body weight changed more than 10% during treatment, recalculation of the dosage is required.

3) Dosage adjustment is allowed according to different chemotherapy drug formulation, but the adjusted range cannot exceed 5% of the calculated dose (95%~105%), except for rituximab.

4) Radiotherapy to any residual mass(es) which were considered to have tumor activity by imaging post-therapy or original sites of disease bulk (> 7.5 cm^2^) after the completion of chemotherapy (CHOP or CEpOP) +/- rituximab was permitted at discretion of the clinical investigator.

5) Rituximab is not mandatory during treatment.

6) For patients with high tumor load or high ratios of lymphocyte, rituximab can be incorporated into chemotherapy after 1-2 courses of treatment in order to prevent acute tumor lysis syndrome.

7) For patients receiving rituximab, as long as hepatitis B surface antigen is positive, antiviral therapy should be received.

8) Prophylactic acid treatment is recommended for older patients and those with gastric disease during glucocorticoid therapy;

9) 5-HT3 inhibitor antiemetic therapy is routinely recommended;

10) Blood glucose control should be monitored in patients with diabetes;

11) Patients with ECOG PS score > 2 or high tumor load can be considered for a non-anthracycline containing regimen (such as vincristine combined with prednisone), and then enrolled after PS scores recover or tumor regression.

**6．Study items**

6.1 laboratory examination

1) Before enrollment: blood routine test, liver and kidney function, β2-microglobulin, LDH, Detection of hepatitis B, syphilis, HIV;

2) During and after treatment, blood routine test, liver and kidney function, β2-microglobulin, LDH

6.2 Examination of imaging

1) Before enrollment: contrast-enhanced computed tomography scan (CT-scan) of neck, thorax, abdomen and pelvis

2) During and after treatment: lesions or suspicious lesion site CT are repeated every 2 cycles and tumor efficacy evaluation at the same time

3) MRI examination (such as nasopharyngeal etc.) according to the lesion

4) Upper gastrointestinal barium meal (GI) examination should be performed in patients with primary Waldeyer's ring (nasopharyngeal tonsil and tongue, and oropharynx). Gastroscopy confirmed in case of abnormal GI examination results.

5) PET/CT examination is performed based on avalibility

6.3 special examination

1) Before enrollment: bone marrow puncture and biopsy;

2) Repeated bone marrow puncture and biopsy should be required for patients with bone marrow involvement

6.4 Heart function examination

6.4.1 Radionuclide angiography (RNA);

1) Before enrollment, Post-4 cycles and Post-6 cycles;

2) After 6 cycles of treatment, repeated every 2 cycles;

3) Performed at any time that abnormal heart function happens during treatment;

4) Repeat is recommended for patients receiving post-chemotherapy mediastinal radiotherapy;

5) Repeat is recommended for recurrent patients

Note: When abnormal LVEF is detected by RNA at any time, confirming the subclinical cardiac toxicity, patients should be withdrawn from the study and discontinue anthracycline therapy

6.4.2 Electrocardiogram (EKG)

1) Before enrollment, before and after each cycle;

2) Performed whenever symptomatic abnormal heart function occurs

6.4.3 Heart function score (NYHA cardiac functional grade)

1) Before enrollment, before and after each cycle;

2) Performed at any time that abnormal heart function happens during treatment;

6.4.4 CHF risk factors

1) Documents of CHF associated high risk factors before enrollment

2) Smoking, obesity (body mass index, >30kg/m^2^), hypertension, diabetes, chronic obstructive pulmonary disease.

6.5 Blood biomarkers examination

1) Before enrollment, before and after each cycle of treatment, blood sample is conserved in the clinical laboratory

2) Performed at any time that abnormal heart function happens during treatment;

3) Detection of troponin T and troponin I and atrial natriuretic peptide (ANP) and brain natriuretic peptide (BNP) at appropriate time

Note: the specific inspection process (Table 1)

**7 dose adjustment**

7.1 Hematology:

1) Based on the most severe hematological toxicity of last cycle;

2) CTC 4 thrombocytopenia, cyclophosphamide and doxorubicin / epirubicin dose are reduced by 20%;

3) CTC 4 leukopenia <3 days, maintain 100% dosage;

4) CTC 4 leukopenia ≥ 3 days or grade 3/4 of febrile neutropenia, cyclophosphamide and doxorubicin / epirubicin dose are reduced by 20%; Prophylactic G-CSF treatment can also be considered (5ug/kg/ day, at least once every 5 days until the ANC ≥ 10 × 10^9^/L)

5) Drug dosage adjustment level is shown in table 1.

| Table 1: Drug dosage adjustment (Hematology) | | | |
| --- | --- | --- | --- |
|  | Cyclophosphamide | Doxorubicin | Epirubicin |
| First adjustment | 600 mg/m^2^ | 40 mg/m^2^ | 56 mg/m^2^ |
| Second adjustment | 480 mg/m^2^ | 32 mg/m^2^ | 44.8 mg/m^2^ |

7.2 Liver function:

1) CTC 3/4 liver function damage, cyclophosphamide and doxorubicin / epirubicin dose are reduced by 25%;

2) Before chemotherapy, total bilirubin, ALT and AST were <1.5 × UNL, maintain 100% dosage;

3) Before chemotherapy, total bilirubin, ALT or AST between 1.5~3.0 × UNL, cyclophosphamide dose are reduced by 25%, vincristine and doxorubicin / epirubicin dose are reduced by 50%;

4) Before chemotherapy, total bilirubin, ALT or AST more than 3 x UNL, delay administration of chemotherapy, receive liver treatment

5) Attending physician can decide whether reduce the dosage of chemotherapy or delay chemotherapy and receive liver treatment case by case

6) Drug dosage adjustment level is shown in table 2

| Table 2: Drug dosage adjustment (Liver Function) | | | |
| --- | --- | --- | --- |
|  | Vincristine | Doxorubicin | Epirubicin |
| First adjustment | 1.05 mg/m^2^ | 37.5 mg/m^2^ | 52.5 mg/m^2^ |
| Second adjustment | 0.79 mg/m^2^ | 28.1 mg/m^2^ | 39.4 mg/m^2^ |

7.3 Peripheral nervous system

1) At least CTC 3 peripheral neurotoxicity, vincristine should be suspended except for any other drugs. In the next cycle, vincristine treatment can be continued after peripheral neurotoxicity recover to lower than CTC 3, the dosage of which are reduced by 25%.

2) At most 2 times of dosage reduction, vincristine treatment should be suspended;

3) Drug dosage adjustment level is shown in table 3.

| Table 3: Drug dosage adjustment（Peripheral nervous system） | |
| --- | --- |
|  | Vincristine |
| First adjustment | 1.05 mg/m^2^ |
| Second adjustment | 0.79 mg/m^2^ |

**8. Combination drugs**

1) Physician can decide whether/when to give the cytokine therapy (including G-CSF, EPO or platelet-derived growth factor) and blood component transfusion case by case.

2) IL-11 (because of cardiac toxicity) is prohibited, blood component transfusion is suggested for patients with serious thrombocytopenia.

3) Routine use of dexrazoxane is prohibited;

4) Prophylactic antibiotic therapy is allowed for patients with previous occurrence of febrile neutropenia.

5) Chinese traditional medicine is not recommended at the same time.

**9 Statistical Analysis**

We calculated that 320 participants (160 per arm) would have 90% power to test the hypothesis that EPI would reduce the incidence of the primary endpoint by 11%, compared with 20% incidence of control group, after 4 cycles of treatment, at a two-sided type I error rate of 0.05. Considering drop-out, 400 patients would need to be enrolled for the study.

All the analyses were performed on an intention-to-treat basis. Continuous variables were expressed as means and standard deviations or medians and interquartile ranges. Categorical variables were expressed as frequencies (%). Student’s t-tests, Wilcoxon signed rank test, Chi-square tester Fisher’s exact test were applied to test the differences between treatment and control, as appropriate. PFS was compared using the Kaplan-Meier method and log-rank test. The Cox proportional hazards model was used to estimate the hazard ratios and associated 95% confidence intervals.

**10．Reference**

1. Morton LM, Wang SS, Devesa SS, et al. Lymphoma incidence patterns by WHO subtype in the United States, 1992-2001. Blood 2006;107:265-276
2. Chau, I, Jones, R, Cunningham, D, et al. ^[1](#_ENREF_1)^?. Br J Cancer 2003; 89:36-42
3. Fisher R, Gaynor E, Dahlberg S, et al. Comparison of a standard regimen (CHOP) with three intensive chemotherapy regimens for advanced non-Hodgkin's lymphoma. N Engl J Med 1993; 328:1002-1006
4. Coiffier B, Lepage E, Briere J, et al. CHOP chemotherapy plus rituximab compared with CHOP alone in elderly patients with diffuse large-B-cell lymphoma. N Engl J Med 2002; 346:235-242
5. Pfreundschuh M, Trumper L, Osterborg A, et al. CHOP-like chemotherapy plus rituximab versus CHOP-like chemotherapy alone in young patients with good-prognosis diffuse large-B-cell lymphoma: a randomised controlled trial by the MabThera International Trial (MInT) Group. Lancet Oncol 2006; 7:379-391
6. Pfreundschuh M, Schubert J, Ziepert M, et al. Six versus eight cycles of bi-weekly CHOP-14 with or without rituximab in elderly patients with aggressive CD20+ B-cell lymphomas: a randomised controlled trial (RICOVER-60). Lancet Oncol 2008; 9:105-116
7. Singal PK, Deally CMR, Weinberg LE.. J Mol Cell Cardiol 1987; 19:817-828
8. Isner JM, Ferrans VJ, Cohen SR, et al. Clinical and morphologic cardiac findings after anthracycline chemotherapy. Analysis of 64 patients studied at necropsy. Am J Cardiol 1983; 51:1167-1174
9. Von Hoff DD, Layard MW, Basa P, et al. Risk factors for doxorubicin-induced congestive heart failure. Ann Intern Med 1979; 91:710
10. Swain SM, Whaley FS, Ewer MS. Congestive heart failure in patients treated with doxorubicin. Cancer 2003; 97:2869-2879
11. Schwartz RG, McKenzie WB, Alexander J, et al. Congestive heart failure and left ventricular dysfunction complication doxorubicin therapy. Seven-year experience using serial radionuclide angiocardiography. Am J Med 1987; 82:1109-1118
12. Mousiainen T, et al. Comparison of echocardiography and radionuclide ventriculography in the follow-up of left ventricular systolic function in adult lymphoma patients during doxorubicin therapy. J Intern Med 2001; 249:297-303
13. Morrow DA, Cannon CP, Jesse RL, et al. National Academy of Clinical Biochemistry Laboratory Medicine Practice Guidelines: Clinical characteristics and utilization of biochemical markers in acute coronary syndromes. Circulation 2007;115:e356–e375
14. Lipshultz SE, Rifai N, Dalton VM, et al. The effect of dexrazoxane on myocardial injury in doxorubicin-treated children with acute lymphoblastic leukaemia. N Engl J Med 2004; 351:145–153
15. Auner HW, Tinchon C, LinkeschW, et al. Prolonged monitoring of troponin T for the detection of anthracycline cardiotoxicity in adults with hematological malignancies. Ann Hematol 2003;82: 218–222
16. Kilickap S, Barista I, Akgul E, et al. 005; 16:798–804
17. Cardinale D, Sandri MT, Martinoni A, et al. Left ventricular dysfunction predicted by early troponin I release after high-dose chemotherapy. J Am Coll Cardiol 2000; 36:517–522
18. Sandri MT, Cardinale D, Zorzino L, et al. Minor increases in plasma troponin I predict decreased left ventricular ejection fraction after high-dose chemotherapy. Clin Chem 2003;49:248–252
19. Wang TJ, Larson MG, Levy D, et al. Plasma natriuretic peptide levels and the risk of cardiovascular events and death.NEngl J Med 2004; 350:655–663
20. Hayakawa H, Komada Y, Hirayama M, et al. Plasma levels of natriuretic peptides in relation to doxorubicin-induced cardiotoxicity and cardiac function in children with cancer. Med Pediatr Oncol 2001; 37:4–9
21. Nousiainen T, Vanninen E, Jantunen E, et al. Natriuretic peptides during the development of doxorubicin-induced left ventricular diastolic dysfunction. J Intern Med 2002; 251:228–234
22. Ryberg M, Nielsen D, Skovsgaard T, et al. Epirubicin cardiotoxicity: an analysis of 469 patients with metastatic breast cancer. J Clin Oncol 1998; 16:3502-3508
23. Launchbury AP, Habboubi N. Epirubicin and doxorubicin: a comparison of their characteristics, therapeutic activity and toxicity. Cancer Treat Rev 1993; 19:197-228
24. Nair R, Ramakrishnan G, Nair NN, et al. A randomized comparison of the efficacy and toxicity of epirubicin and doxorubicin in the treatment of patients with non-Hodgkin's lymphoma. Cancer 1998; 82: 2282-2288
25. Lambertenghi Deliliers G, Butti C, Baldini L, et al. A cooperative study of epirubicin with cyclophosphamide, vincristine and prednisone (CEPOP) in non-Hodgkin's lymphoma. Haematologica 1995; 80:318-324
26. Chim CS, Kwong YL, Lie AK, et al. CEPOP treatment results and validity of the International Prognostic Index in Chinese patients with aggressive non-Hodgkin's lymphoma. Hematol Oncol 1998; 16:117-123
27. Basaran M, Bavbek ES, Sakar B, et al. Treatment of aggressive non-Hodgkin's lymphoma with dose-intensified epirubicin in combination of cyclophosphamide, vincristine, and prednisone (CEPOP-100): a phase II study. Am J Clin Oncol 2001; 24:570-575
28. Economopoulos T, Dimopoulos MA, Mellou S, et al. Treatment of intermediate- and high-grade non-Hodgkin's lymphoma using CEPOP versus CNOP. Eur J Haematol 2002; 68:135-143
29. Economopoulos T, Psyrri A, Dimopoulos MA, et al. CEPOP-21 versus CEPOP-14 chemotherapy with or without rituximab for the first-line treatment of patients with aggressive lymphomas: results of the HE22A99 trial of the Hellenic Cooperative Oncology Group. Cancer J 2007; 13:327-334
